# A two-stage unified Bayesian framework for adolescent physical fitness surveillance in Sichuan, China: Latent factor measurement followed by spatiotemporal modeling

**DOI:** 10.1371/journal.pone.0355280

**Published:** 2026-08-11

**Authors:** Honglei Wei, Haoran Li, Maowu Pu, Zhou He, Yiru Wang

**Affiliations:** 1 School of Mathematics, Southwest Jiaotong University, Chengdu, Sichuan, China; 2 Sichuan Province Big Data Research and Joint Application Technology Center of Student Health, Chengdu, Sichuan, China; 3 College of International Education, Chengdu University of Technology (Oxford Brookes College), Chengdu, Sichuan, China; Zhejiang Agriculture and Forestry University: Zhejiang A and F University, CHINA

## Abstract

Large-scale adolescent fitness testing produces heterogeneous multi-item measurements. Translating these measurements into interpretable and comparable evidence for provincial surveillance remains challenging. We propose a two-stage Bayesian framework for adolescent physical fitness surveillance in Sichuan, China. In Stage 1, Bayesian confirmatory factor analysis maps cross-grade test items onto four latent fitness factors: strength, speed, endurance, and flexibility. This stage explicitly models item-level measurement error and item heterogeneity. In Stage 2, factor-level outcomes are modeled using Bayesian spatiotemporal hierarchical regression. This stage estimates covariate associations, temporal trends, and residual spatial heterogeneity.

In the Sichuan application, the four latent factors showed clear and interpretable spatial structure. Residual spatial clustering persisted after adjustment for covariates, educational-stage effects, and shared temporal trends. Urbanization rate showed positive conditional associations with all four factors. Selected socioeconomic, geographic, and environmental covariates showed domain-specific associations, such as negative associations of GDP per capita and population density mainly with strength and speed. Model comparison using PSIS-LOO indicated that models with spatial random effects provided better conditional pointwise predictive fit than corresponding no-spatial models for all four factors, with satisfactory Pareto-*k* diagnostics.

By separating latent measurement from regional spatiotemporal modeling, the proposed framework supports more interpretable regional comparisons. It also helps identify regions with persistent residual advantages or disadvantages, providing quantitative evidence for targeted monitoring and policy discussion.

## Introduction

Adolescent physical fitness is a multidimensional marker of youth health. It includes cardiorespiratory fitness, muscular strength and power, flexibility, and body composition. Physical fitness in youth is associated with later-life metabolic and cardiovascular risks and mortality [1,2]. However, insufficient physical activity remains widespread among adolescents [3]. Secular trends in cardiorespiratory fitness also vary across countries and regions [4]. These findings highlight the need for school- and community-based surveillance and intervention strategies [5].

For provincial surveillance, overall averages are insufficient. Policy-relevant questions concern which regions and educational stages are persistently disadvantaged, whether these disparities cluster geographically, and whether they remain after adjustment for socioeconomic and environmental correlates. Related adolescent health indicators, such as BMI and overweight/obesity prevalence, show clear spatial heterogeneity and temporal non-stationarity in both global and China-specific studies [6,7]. Ignoring spatial dependence and temporal dynamics may bias effect estimates, underestimate uncertainty, and misattribute residual spatial clustering to observed covariates [8].

Fitness testing data introduce an additional measurement challenge. School surveillance systems collect multiple test items that jointly reflect latent fitness domains. These items also contain item-specific measurement error and correlations. In addition, item batteries differ across educational stages, which may weaken cross-grade comparability. Existing studies often analyze total scores or selected items. They rarely model the latent multi-item structure and grade-specific item availability within a unified framework. If raw item scores are directly aggregated and treated as error-free outcomes, measurement error may be transferred into downstream spatial random effects. This can destabilize spatial maps and bias covariate inference [9,10]. The central methodological challenge is therefore to construct comparable latent fitness representations under heterogeneous item batteries and then conduct spatiotemporal inference with uncertainty quantification.

We propose a two-stage Bayesian framework for provincial adolescent fitness surveillance. In Stage 1, Bayesian confirmatory factor analysis maps heterogeneous test items onto four latent fitness domains while accounting for measurement error and item heterogeneity. In Stage 2, factor-level outcomes are modeled using a Bayesian spatiotemporal hierarchical model with covariates, structured spatial effects, and temporal dynamics [11–13]. We assess the empirical contribution of spatial structure by comparing models with and without spatial random effects using predictive criteria such as PSIS-LOO.

The main contributions are threefold. First, the proposed measurement-first strategy links multi-item fitness testing data to interpretable latent domains and reduces cross-grade incomparability. Second, factor-level spatiotemporal modeling improves the interpretation and stability of regional fitness surveillance. Third, the Bayesian framework provides uncertainty quantification and a basis for future spatially varying coefficient or non-stationary extensions [14–16].

The remainder of the paper is organized as follows. Section 1 reviews related evidence and methodological developments. Section 2 describes the data, model specifications, priors, and inference. Section 3 presents the factor structure, covariate effects, spatial maps, and LOO-based model comparison. Section 4 discusses implications for public health and education governance. Section 5 concludes with limitations and future work.

## 1 Literature review

This section motivates the proposed two-stage framework from three perspectives: latent measurement of multidimensional fitness, Bayesian spatiotemporal inference for regional surveillance, and model evaluation for spatial structure.

### 1.1 Measurement of multi-dimensional fitness: Latent structure, error, and comparability

Physical fitness is commonly viewed as a multidimensional construct. It includes cardiorespiratory fitness, muscular strength and power, flexibility, and body composition [1,2]. In school-based surveillance, these dimensions are measured through multiple test items. These items share latent fitness information, but they also contain item-specific measurement error. Treating aggregated item scores as error-free outcomes may confound measurement error with regional heterogeneity and weaken interpretability in downstream regression or spatial models [9,10].

Cross-grade comparability is another challenge. Testing batteries differ across educational stages, so apparent differences may reflect changes in measurement rather than differences in latent fitness. Bayesian SEM/CFA provides a useful framework for modeling measurement error and linking heterogeneous item sets through a common latent structure. This literature provides the methodological basis for Stage 1.

### 1.2 Bayesian spatiotemporal inference for regional surveillance: Disease mapping and small-area estimation

Bayesian disease mapping and small-area estimation are widely used in regional public health surveillance. Hierarchical models with structured spatial effects, such as CAR/BYM priors, and temporal components, such as random walks or AR(1) processes, can stabilize small-area estimates and quantify uncertainty [8,11]. Improved parameterizations, such as BYM2, provide clearer decompositions of spatial variation and improve interpretability [12]. INLA offers efficient approximate Bayesian inference for latent Gaussian models and is widely used in spatiotemporal disease mapping [13]. Modern priors, including penalized complexity priors, further support parsimonious and reproducible modeling [17].

These approaches are mature for outcomes such as obesity prevalence [6,7,18]. However, multi-item fitness surveillance introduces an upstream outcome-construction problem. If latent structure and comparability are not addressed first, spatial random effects may partly absorb measurement artifacts. This motivates a measurement-first, inference-second strategy.

### 1.3 Spatial heterogeneity: From GWR/MGWR to Bayesian spatially varying coefficients

Global regression models assume spatially homogeneous covariate effects. This assumption may be restrictive when relationships vary across locations. GWR and MGWR estimate local coefficients and allow different spatial scales across covariates [19–21]. Bayesian spatially varying coefficient processes provide a probabilistic alternative. They quantify uncertainty and can be modeled jointly with spatially correlated residual structure [14–16]. These developments suggest that provincial surveillance models should retain a structure that can be extended to spatially heterogeneous associations.

### 1.4 Model evaluation in hierarchical Bayesian spatiotemporal models

Model comparison is needed to assess whether structural components, such as spatial random effects, are empirically useful. PSIS-LOO provides a widely used criterion for Bayesian predictive comparison, and Pareto-*k* diagnostics assess the stability of the importance sampling approximation [22]. In regional surveillance, comparing models with and without spatial random effects helps evaluate whether residual spatial structure contributes predictive information and affects the interpretation of spatial inequality.

### 1.5 Summary and motivation

Existing studies provide strong foundations for latent measurement modeling and spatiotemporal surveillance. However, provincial multi-grade fitness testing data still involve three linked challenges: latent structure, measurement error, and cross-grade item heterogeneity. The proposed Stage 1–Stage 2 Bayesian framework addresses these challenges by first constructing interpretable latent fitness factors and then modeling their regional spatiotemporal patterns with uncertainty quantification.

## 2 Materials and methods

### 2.1 Study setting, data source, and observational unit

This study analyzes student physical fitness testing data from Sichuan Province, China. The spatial unit is the prefecture-level city, indexed by i=1,...,N. The study period spans 2019–2024 and is aggregated annually, indexed by t=1,...,T. Because the testing protocol and item set vary by educational stage, we define the basic observational unit as a *city* × *educational stage* × *year* cell, denoted by (*i*,*g*,*t*). Here, g=1,...,G indexes five educational stages: Primary grades 1–2, Primary grades 3–4, Primary grades 5–6, middle school, and high school.

The raw data are recorded at the student level and include multiple physical fitness item scores on a 0–100 scale. Study coverage and the distribution of city–educational stage–year cell sizes are summarized in Table 1. These differences are defined by the standardized testing protocol and should be distinguished from ordinary item nonresponse. Directly modeling item-level outcomes across all stages without accounting for this protocol heterogeneity may introduce structural bias. We therefore adopt a two-step strategy. Stage 1 extracts latent fitness factors from item-level scores using a Bayesian measurement model. Stage 2 fits a provincial spatiotemporal hierarchical model to the factor-level outcomes, enabling unified inference across cities, years, and educational stages.

**Table 1:** Summary of study coverage, design, and cell-size distribution.

Component	Description
Spatial units (cities/prefectures)	21
Study period (years)	2019–2024
Number of years	6
Educational stages	Primary 1–2, Primary 3–4, ...
Number of educational stages	5
Physical fitness test items	BMI, vital capacity, 50 m sprint, ...
Number of test items	11
Total student-level records	57456015
Missingness among administered item records	0%
**Distribution of city–stage–year cell sizes**	
Number of city–stage–year cells	630
Minimum cell size	15,071
25th percentile	46,200
Median	67,500
Mean	91,200
75th percentile	103,275
Maximum cell size	511,756
Cells with fewer than 10,000 records	0

Table notes: *i* denotes city/prefecture, *g* denotes educational stage, and *t* denotes year. A city–stage–year cell refers to one city/prefecture, one educational stage, and one calendar year. Cell size denotes the number of student-level records contributing to the corresponding aggregated factor score. Missingness among administered item records refers to items that were expected to be tested under the standardized testing protocol. Structural non-administration refers to items not included for a given educational stage by design and is not counted as ordinary missingness.

Age- or grade-related differences are represented through the five educational stages, which correspond to the age- and stage-specific testing protocol. These educational-stage differences are further adjusted in Stage 2 through the factor-specific educational-stage effects $\delta_{g,k}$. Sex-specific latent factor structures are not modeled separately because the present study focuses on regional surveillance at the city–educational stage–year level rather than individual-level sex-specific inference. The analysis uses standardized 0–100 item scores from the official fitness testing system, and the resulting scores are mapped onto latent factors without stratifying by sex.

### 2.2 Measures: Test items, latent factors, and covariates

Let *s* index students or individual records and p∈{1,...,J} index test items, with *J* = 11 in this study. The observed outcome is the item score $y_{s,p}$. In Stage 1, Bayesian confirmatory factor analysis (Bayesian CFA) is used to extract four latent fitness factors: *strength*, *speed*, *endurance*, and *flexibility*. These factors are not directly observed test scores. Instead, they summarize shared information from related test items.

The four latent fitness factors correspond to four domains of the five fundamental physical qualities described in the National Physical Exercise Standard Work Guidance Manual. According to the age- and stage-specific definitions of test items in this manual, the observed physical fitness test scores are mapped onto four latent domains. BMI and vital capacity are treated as global health-related indicators because they may reflect multiple aspects of physical fitness rather than a single domain. They are therefore allowed to cross-load on multiple factors. Agility is not modeled as a separate factor because the available adolescent fitness testing protocol does not include a direct agility-related test item.

**Fig 1:**
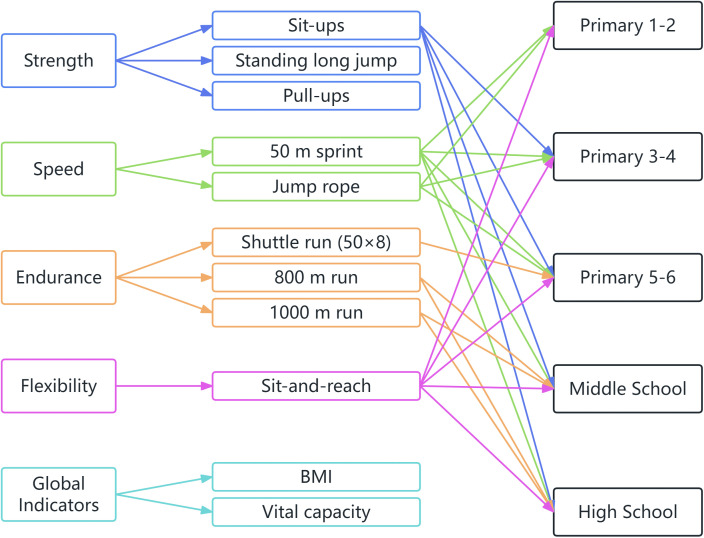
Measurement-linking structure across educational stages. The diagram shows the links among latent fitness domains, administered test items, and educational stages.

Fig 1 illustrates the measurement-linking structure across educational stages. The figure shows how each latent fitness domain is connected to the administered test items and how these items link different educational stages. The administered items are not identical across educational stages. Speed and flexibility are directly linked across stages through the 50 m sprint and sit-and-reach, respectively, both of which are administered in all stages. Strength and endurance have more indirect links because several of their primary items are stage-specific; their cross-stage comparability relies on overlapping items, the common factor structure, and the auxiliary information from BMI and vital capacity. Therefore, the resulting comparisons are interpreted at the latent-domain level rather than as evidence of strict item-level measurement invariance.

**Fig 2:**
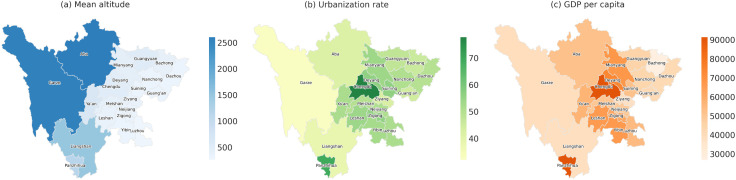
Spatial distribution of selected regional covariates in Sichuan Province. Panels show (A) mean elevation, (B) urbanization rate, and (C) GDP per capita. Administrative boundary data were obtained from gbOpen under the CC BY 4.0 License. The data were subset to Sichuan Province and formatted by the authors for visualization.

To explain regional variation in fitness factors, Stage 2 incorporates an annual city-level covariate vector ${𝐱}_{i,t}\in {\mathbb{R}}^{8}$. Covariates were obtained from official statistical yearbooks and meteorological sources and aligned by city and year. All covariates were Z-score standardized (mean 0, standard deviation 1) prior to modeling to improve coefficient comparability and computational stability. Definitions and processing details are provided in Table 2; examples of spatial patterns are shown in Fig 2.

**Table 2:** Regional-level covariates used in the spatiotemporal model.

Covariate	Unit	Level
GDP per capita	CNY per person	City-year
Urbanization rate	%	City-year
Population density	Persons per km^2^	City-year
Culture, sports, and media expenditure	10^4^ CNY	City-year
Mean elevation	m	City
Annual mean temperature	°C	City-year
Annual precipitation	mm	City-year
Annual mean relative humidity	%	City-year

Table notes: All covariates were obtained from official statistical yearbooks and meteorological sources and aligned at the city-year level unless otherwise specified.

Spatial dependence is defined by administrative contiguity: $w_{ij}=1$ if two cities share a boundary and $w_{ij}=0$ otherwise, yielding a symmetric adjacency matrix $W=[w_{ij}]$ and a degree matrix $D=diag(\sum_{j}w_{ij})$.

### 2.3 Overview of the two-step Bayesian framework

**Fig 3:**
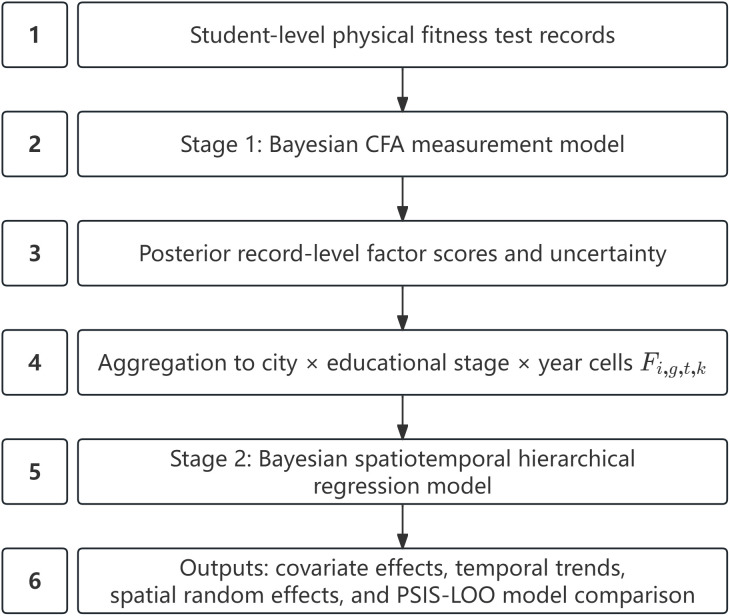
Workflow of the proposed two-step Bayesian framework.

The proposed framework consists of two connected stages: a measurement stage followed by a spatiotemporal modeling stage (Fig 3). The first stage addresses the latent structure, item-level measurement error, and stage-specific item availability in multi-item fitness testing data. The second stage uses the resulting factor-level outcomes for provincial spatiotemporal surveillance.

In Stage 1, Bayesian confirmatory factor analysis maps observed item scores onto four latent fitness domains and produces posterior distributions of record-level factor scores. These posterior factor scores are then aggregated within each city–educational stage–year cell, denoted by (*i*,*g*,*t*), to obtain factor-level observations $F_{i,g,t,k}$ for factor k=1,...,4. Cell-level posterior uncertainty, such as posterior standard deviations, is retained and propagated to Stage 2.

In Stage 2, separate Bayesian spatiotemporal hierarchical models are fitted for the four latent factors. Each model estimates covariate associations, educational-stage effects, temporal trends, and structured spatial random effects. Models with and without the spatial component are further compared using PSIS-LOO to assess the incremental predictive value of explicitly modeling spatial dependence.

### 2.4 Stage 1: Bayesian CFA mapping from test items to four factors

Stage 1 maps multi-item fitness scores to four interpretable latent fitness factors: *strength*, *speed*, *endurance*, and *flexibility*. Conceptually, each observed test score is treated as a noisy indicator of one or more underlying fitness domains. The purpose of the measurement model is to summarize the shared information across related test items into factor scores that can be compared and modeled at the regional level.

Let *s* index student records, *g*(*s*) denote the educational stage of record *s*, and p∈{1,...,J} index test items (*J* = 11). The observed item score is $y_{s,p}$. Let ${𝐅}_{s}=(F_{s,1},...,F_{s,4}{)}^{⊤}\in {\mathbb{R}}^{4}$ denote the latent factor vector, $\alpha_{p}$ the item intercept, $\lambda_{p}=(\lambda_{p,1},...,\lambda_{p,4}{)}^{⊤}$ the item loading vector, and $\sigma_{y,p}$ the item-specific residual standard deviation.

To represent the stage-specific testing protocol, let $A_{g,p}$ be the item-administration indicator, where $A_{g,p}=1$ if item *p* is administered in educational stage *g* and $A_{g,p}=0$ otherwise. For administered items, the Bayesian CFA measurement model is


$$\begin{array}{c} y_{s,p}|A_{g(s),p}=1\sim 𝒩\;\left(\alpha_{p}+\lambda_{p}^{⊤}{𝐅}_{s},\;\sigma_{y,p}^{2}\right). \end{array}$$ (1)

Items with $A_{g(s),p}=0$ are structurally not administered under the testing protocol and therefore do not contribute to the likelihood.

This specification uses a common latent-factor orientation across educational stages. The measurement parameters $\alpha_{p}$, $\lambda_{p}$, and $\sigma_{y,p}$ are item-specific and are shared across the educational stages in which item *p* is administered. Educational-stage dependence enters Stage 1 through the administration matrix $A_{g,p}$, which determines the set of observed items contributing to the likelihood. Thus, the model is a common-orientation Bayesian CFA with stage-specific item administration, rather than a multi-group CFA with grade-specific loadings or intercepts.

For identification and scale anchoring, the latent factors are assigned standard normal priors. Factor loadings are specified using theory-guided structured priors: primary item–factor loadings are centered on positive values to stabilize factor orientation, non-primary cross-loadings are shrunk toward zero, and BMI and vital capacity are allowed to load more freely on all factors as global health-related indicators. Cross-stage comparability is therefore interpreted at the latent-domain level and is supported by the shared four-domain structure, common anchor items, overlapping item structures, and structured loading priors. Stage-specific differences in factor levels are further adjusted in Stage 2 through educational-stage effects.

#### Computation and outputs.

Stage 1 was implemented in PyMC using the No-U-Turn Sampler (NUTS) with four chains. Convergence and sampling stability were assessed using standard MCMC diagnostics, including $\hat{R}$, effective sample size (ESS), and divergent transitions. Posterior means of record-level factor scores were then aggregated within each city–educational stage–year cell (*i*,*g*,*t*) to form factor-level observations $F_{i,g,t,k}$. Cell-level pos*t*erior uncertainty, such as posterior standard deviations, was retained and propagated to Sta*g*e 2.

### 2.5 Stage 2: Bayesian spatiotemporal model on factor observations

Stage 2 fits Bayesian spatiotemporal regression models to the factor-level outcomes obtained from Stage 1. For interpretability, separate single-factor models are fitted for the four latent fitness factors. Each model decomposes the factor-level outcome into covariate associations, educational-stage effects, residual spatial structure, and annual temporal variation.

Let $F_{i,g,t,k}$ denote the aggregated factor score for city *i*, educational stage *g*, year *t*, and factor k∈{1,2,3,4}. Let ${SE}_{i,g,t,k}$ denote the corresponding uncertainty measure obtained from Sta*g*e 1. To propagate measurement uncertainty, the observation model is specified as


$$\begin{array}{c} F_{i,g,t,k}\sim 𝒩\;\left(\mu_{i,g,t,k},\;\sigma_{obs,k}^{2}+{SE}_{i,g,t,k}^{2}\right), \end{array}$$ (2)

where $\sigma_{obs,k}^{2}$ captures residual cell-level variability not explained by covariates or random effects.

The linear predictor is


$$\begin{array}{c} \mu_{i,g,t,k}=\alpha_{k}+{𝐱}_{i,t}^{⊤}\beta_{k}+\delta_{g,k}+S_{i,k}+T_{t,k}. \end{array}$$ (3)

Here, $\alpha_{k}$ is the factor-specific intercept, $\beta_{k}$ represents factor-specific associations with standardized city-year covariates, $\delta_{g,k}$ denotes the educational-stage effect for factor *k*, $S_{i,k}$ is the residual spatial effect for city *i*, and $T_{t,k}$ captures annual temporal variation. The $\delta_{g,k}$ adjusts for systematic differences in the mean level of each latent factor across educational stages after Stage 1 factor extraction. Therefore, the covariate effects $\beta_{k}$, spatial effects $S_{i,k}$, and temporal effects $T_{t,k}$ are interpreted conditional on educational-stage differences.

To reduce confounding with the intercept, the educational-stage effects are centered:


$$\begin{array}{c} \delta_{g,k}=\delta_{g,k}^{*}-\frac{1}{G}\sum_{g^{\prime }=1}^{G}\delta_{g^{\prime },k}^{*}. \end{array}$$ (4)

The spatial effects are assigned a proper conditional autoregressive (CAR) prior based on city adjacency. This prior encourages neighboring cities to have similar residual effects while allowing local deviations. Let *W* denote the symmetric adjacency matrix and $D=diag(\sum_{j}w_{ij})$ the degree matrix. In compact form,


$$\begin{array}{c} {𝐒}_{k}\sim 𝒩\;\left(0,\;(\tau_{k}Q(\rho ){)}^{-1}\right),\;Q(\rho )=D-\rho W. \end{array}$$ (5)

The spatial dependence parameter ρ is constrained to its admissible range to ensure a proper prior. The spatial effects are also centered to reduce collinearity with the intercept.

The temporal component is modeled using a first-order Gaussian random walk:


$$\begin{array}{c} T_{t,k}=T_{t-1,k}+\eta_{t,k}, \end{array}$$ (6)

which captures gradual year-to-year changes shared across cities.

For each latent factor, the Stage 2 model was fitted in PyMC using NUTS. Standard convergence and sampling diagnostics were examined to assess sampling stability. To evaluate the empirical contribution of spatial structure, a no-spatial model was also fitted by removing $S_{i,k}$ and compared with the full model using PSIS-LOO. Pareto-*k* diagnostics were used to assess the reliability of the importance sampling approximation, and posterior predictive checks were used to examine model adequacy. For readability, the prior distributions used in both Stage 1 and Stage 2 are summarized in Table 3, rather than being repeated separately throughout the model description.

**Table 3:** Prior specifications used in the two-stage Bayesian framework.

Stage	Parameter	Prior specification
Stage 1	Latent factor vector ${𝐅}_{s}$	$𝒩(0,I_{4})$
Stage 1	Item intercept $\alpha_{p}$	$𝒩(70,20^{2})$
Stage 1	Item residual standard deviation $\sigma_{y,p}$	HalfNormal(10)
Stage 1	Primary loading $\lambda_{p,k}$	$𝒩(1,0.2^{2})$
Stage 1	Non-primary cross-loading $\lambda_{p,k}$	$𝒩(0,0.1^{2})$
Stage 1	BMI/vital-capacity loading $\lambda_{p,k}$	$𝒩(0,1^{2})$
Stage 2	Residual standard deviation $\sigma_{obs,k}$	HalfNormal(1)
Stage 2	Intercept $\alpha_{k}$	$𝒩(0,2^{2})$
Stage 2	Covariate coefficient $\beta_{k,j}$	$𝒩(0,2^{2})$
Stage 2	Uncentered educational-stage effect $\delta_{g,k}^{*}$	𝒩(0,1)
Stage 2	Spatial precision $\tau_{k}$	Gamma(2,1)
Stage 2	Spatial dependence parameter ρ	Beta(2,2)
Stage 2	Temporal innovation $\eta_{t,k}$	$𝒩(0,\sigma_{T,k}^{2})$
Stage 2	Temporal innovation standard deviation $\sigma_{T,k}$	HalfNormal(0.5)
Stage 2	Initial temporal effect *T*_1,*k*_	𝒩(0,1)

## 3 Results

### 3.1 Stage 1 results

In Stage 1, Bayesian confirmatory factor analysis (Bayesian CFA) mapped multiple physical fitness test item scores to four latent fitness factors: strength, speed, endurance, and flexibility. The model yielded posterior distributions for the loading matrix Λ, item-specific residual standard deviations $\sigma_{y,p}$, and record-level factor scores ${𝐅}_{s}$. Overall, the posterior loading pattern was consistent with the prespecified theory-guided item–factor mapping. Each factor showed a clear set of primary loading items, supporting subsequent spatiotemporal modeling at the factor level.

**Fig 4:**
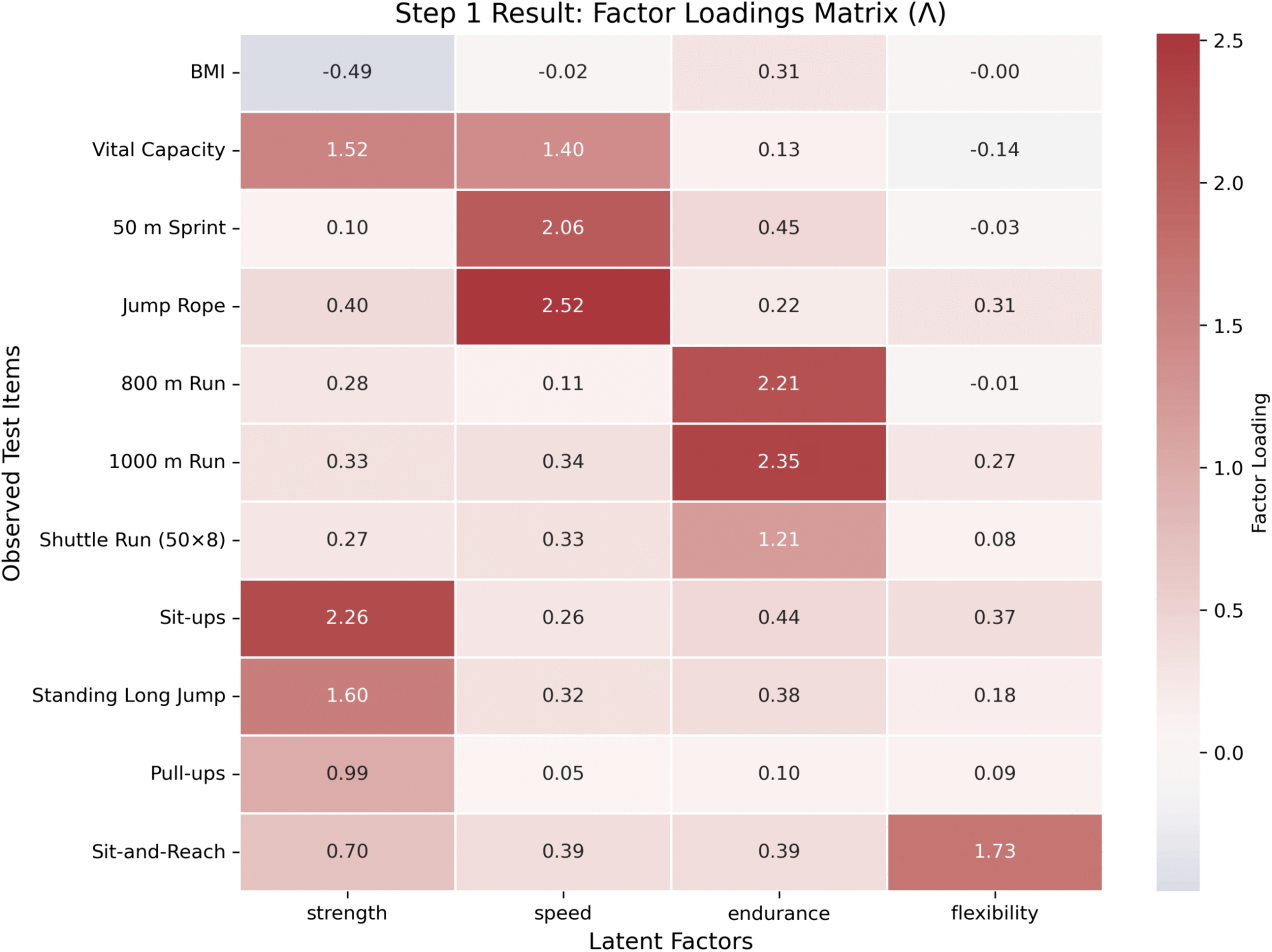
Stage 1 result: posterior mean factor loadings matrix (Λ).

**Loading structure and interpretability.** Fig 4 shows the posterior mean factor-loading matrix. The main trend is a clear block-like loading pattern: most test items loaded most strongly on their prespecified primary fitness domain, while non-primary cross-loadings were generally weaker. The strength factor was mainly driven by sit-ups (posterior mean ≈2.26), standing long jump (≈1.60), and pull-ups (≈0.99). The speed factor was mainly driven by the 50 m sprint (≈2.06) and jump rope (≈2.52). The endurance factor was mainly driven by the 800 m run (≈2.21), 1000 m run (≈2.35), and shuttle run (50×8) (≈1.21). The flexibility factor was mainly driven by sit-and-reach (≈1.73). These patterns support the interpretability of the four-factor measurement structure.

**Global indicators and cross-loadings.** BMI and vital capacity were treated as global health-related indicators and were allowed to load on all four factors. Posterior estimates indicated that BMI loaded negatively on strength (≈−0.49) and positively on endurance (≈0.31), with loadings close to zero for speed and flexibility. Vital capacity showed relatively strong positive loadings on strength and speed (≈1.52 and ≈1.40, respectively). Several other items showed weak-to-moderate cross-loadings, such as the 50 m sprint loading partly on endurance and sit-and-reach loading weakly on non-flexibility factors. These results suggest that the four domains are distinguishable but still share some common fitness-related variation.

**Fig 5:**
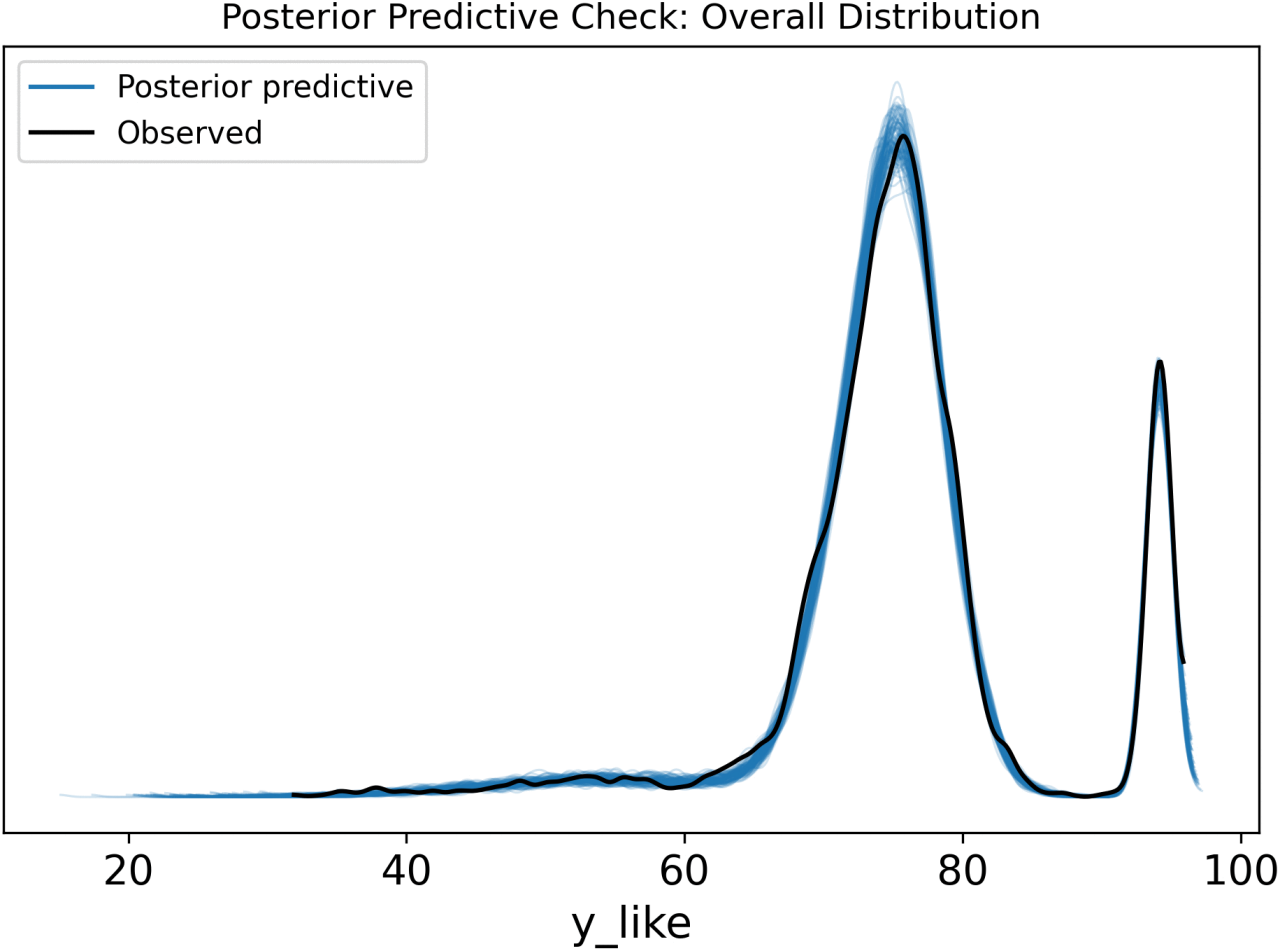
Posterior predictive check (PPC) for Stage 1: overall distribution of observed scores versus posterior predictive draws.

**Posterior predictive checks.** Fig 5 presents the posterior predictive check for the measurement model. The posterior predictive distribution closely followed the observed score distribution. It reproduced the main peak around 75 and the high-score peak around 95 reasonably well. This indicates that the Gaussian measurement model captured the main distributional pattern of the item scores. We also examined the exact boundaries of the 0–100 score scale. The observed proportions of exact 0 and exact 100 scores were both 0%, and the corresponding posterior predictive proportions were also 0%. Thus, the data did not show a substantial point-mass floor or ceiling effect at the score boundaries. Although the scores are bounded by design, the PPC suggests that the Gaussian measurement model provides an adequate approximation in this application.

**Fig 6:**
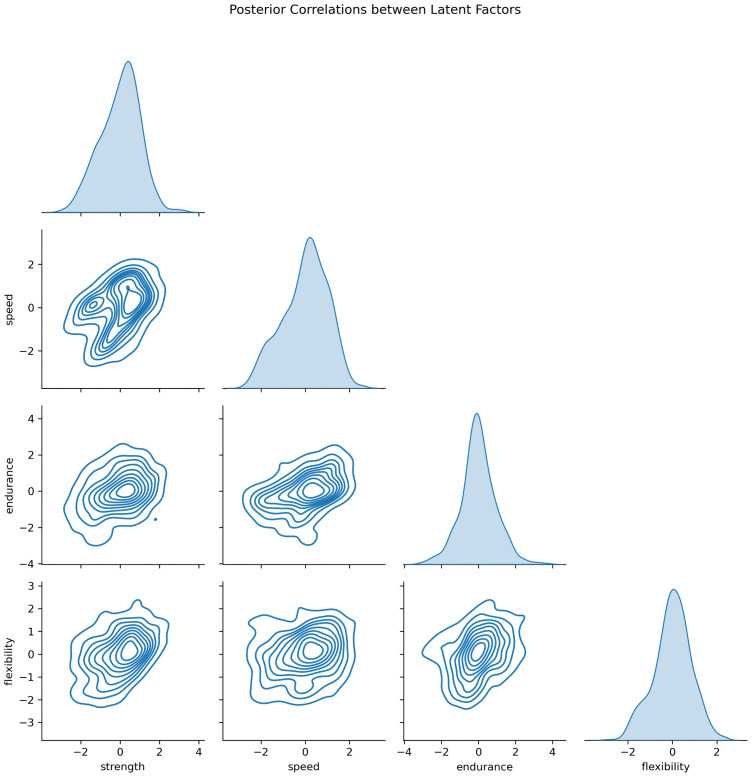
Posterior correlations between latent factors inferred from Stage 1.

**Posterior correlations among factors.** Fig 6 summarizes the posterior correlations among latent factors. Strength, speed, and endurance were positively correlated, and flexibility also showed positive but weaker correlations with the other factors. This pattern suggests a shared general fitness component. At the same time, the correlations were not so high as to collapse the four domains into a single dimension, supporting separate factor-level modeling in Stage 2.

Overall, Stage 1 produced an interpretable four-factor measurement structure. The primary loading patterns supported the intended mapping between test items and the latent domains of strength, speed, endurance, and flexibility. BMI and vital capacity provided additional cross-domain information as global health-related indicators. The posterior predictive checks showed that the Gaussian measurement model captured both the central peak and the high-score peak of the observed score distribution. The proportions of exact 0 and exact 100 scores were 0% in both the observed and posterior predictive data, indicating no substantial point-mass floor or ceiling effect at the score boundaries. The posterior factor correlations showed shared fitness-related variation while preserving sufficient domain-specific distinction for subsequent spatiotemporal modeling.

### 3.2 Stage 2 results

**Fig 7:**
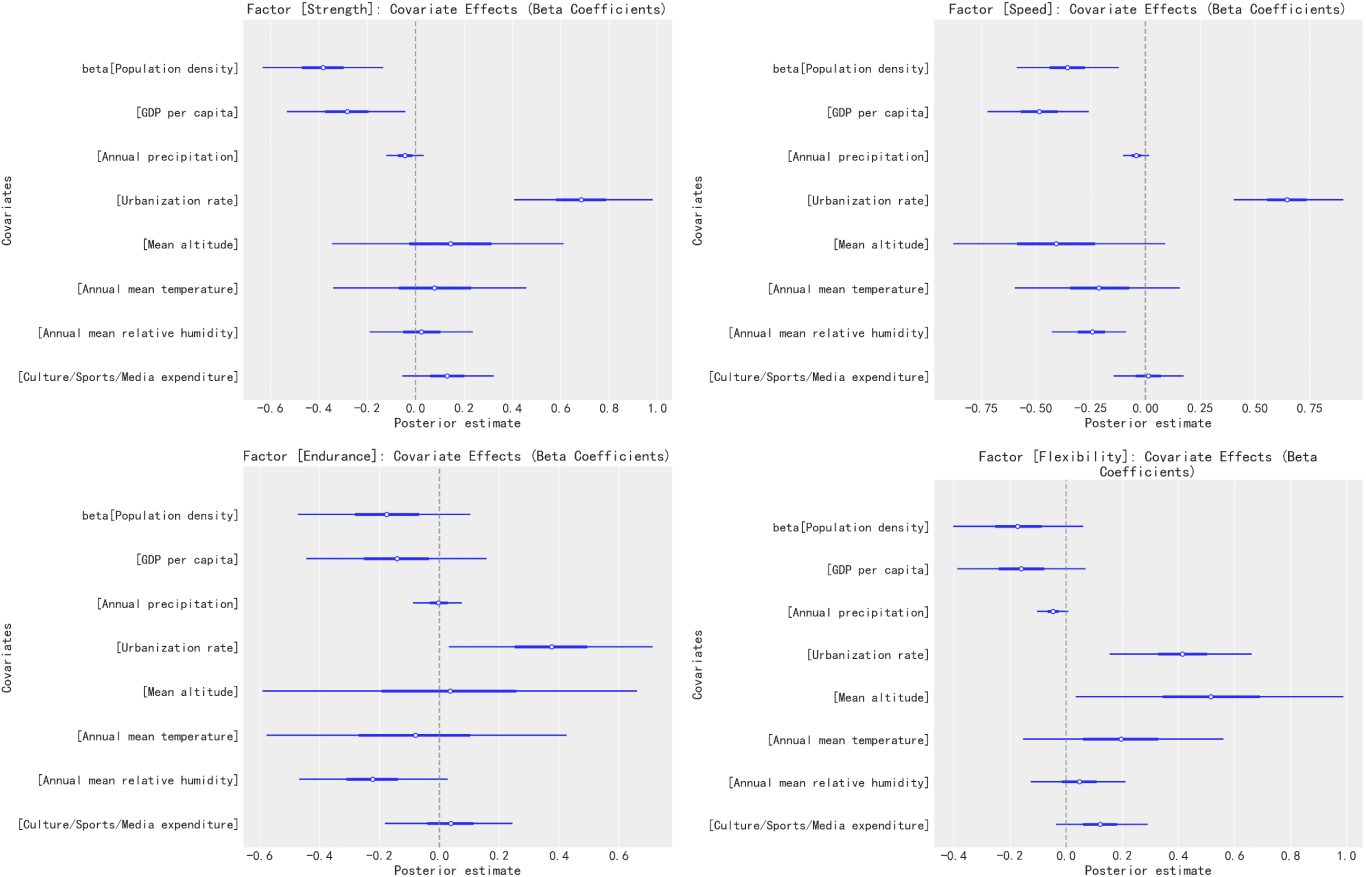
Posterior estimates of covariate effects on the four latent fitness factors. Points indicate posterior means and horizontal bars indicate 94% HDIs (3%–97%). All covariates were standardized.

In Stage 2, Bayesian spatiotemporal hierarchical regression models were fitted to the factor-level outcomes. The aim was to estimate associations between regional socioeconomic, geographic, and climatic covariates and each latent fitness factor. Because all covariates were Z-score standardized before modeling, each coefficient β represents the expected change in the latent factor score associated with a one-standard-deviation increase in the corresponding covariate, conditional on the other model components. We report posterior means and 94% highest density intervals (HDIs; 3%–97% quantiles). Associations whose 94% HDIs did not include 0 were interpreted as having relatively strong posterior support. Full estimates are provided in Table 4, with visual summaries in Fig 7.

**Table 4:** Posterior estimates of covariate effects (β) for four latent fitness factors.

Factor	Covariate	Mean	HDI 3%	HDI 97%	$\hat{R}$	ESS (bulk)
**Strength**						
	Population density (persons per km^2^)	−0.383	−0.632	−0.133	1.003	3914
	GDP per capita (CNY per person)	−0.285	−0.531	−0.042	1.001	4074
	Annual precipitation (mm)	−0.044	−0.121	0.033	1.001	4894
	Urbanization rate (%)	0.684	0.408	0.980	1.000	3580
	Mean elevation (m)	0.147	−0.345	0.612	1.000	2554
	Annual mean temperature (°C)	0.081	−0.342	0.459	1.001	2725
	Annual mean relative humidity (%)	0.026	−0.190	0.237	1.000	3237
	Culture, sports, and media expenditure (10^4^ CNY)	0.130	−0.055	0.323	1.004	4143
**Speed**						
	Population density (persons per km^2^)	−0.359	−0.589	−0.123	1.000	3751
	GDP per capita (CNY per person)	−0.487	−0.722	−0.260	1.001	3872
	Annual precipitation (mm)	−0.043	−0.103	0.016	1.000	4464
	Urbanization rate (%)	0.645	0.404	0.904	1.002	3565
	Mean elevation (m)	−0.410	−0.878	0.091	1.002	2931
	Annual mean temperature (°C)	−0.213	−0.598	0.156	1.001	3054
	Annual mean relative humidity (%)	−0.247	−0.428	−0.088	1.001	3433
	Culture, sports, and media expenditure (10^4^ CNY)	0.012	−0.145	0.175	1.000	3855
**Endurance**						
	Population density (persons per km^2^)	−0.176	−0.473	0.104	1.001	3991
	GDP per capita (CNY per person)	−0.144	−0.444	0.158	1.001	4429
	Annual precipitation (mm)	−0.003	−0.088	0.075	1.000	4801
	Urbanization rate (%)	0.373	0.031	0.712	1.000	3982
	Mean elevation (m)	0.031	−0.590	0.661	1.001	3143
	Annual mean temperature (°C)	−0.084	−0.578	0.425	1.000	3394
	Annual mean relative humidity (%)	−0.225	−0.468	0.027	1.001	3352
	Culture, sports, and media expenditure (10^4^ CNY)	0.038	−0.182	0.244	1.000	3896
**Flexibility**						
	Population density (persons per km^2^)	−0.170	−0.401	0.062	1.000	3963
	GDP per capita (CNY per person)	−0.160	−0.388	0.070	1.001	3652
	Annual precipitation (mm)	−0.046	−0.103	0.008	1.001	4278
	Urbanization rate (%)	0.416	0.155	0.662	1.002	3494
	Mean elevation (m)	0.517	0.035	0.988	1.001	2262
	Annual mean temperature (°C)	0.196	−0.153	0.561	1.001	2344
	Annual mean relative humidity (%)	0.046	−0.126	0.212	1.000	2862
	Culture, sports, and media expenditure (10^4^ CNY)	0.121	−0.035	0.291	1.001	3705

The main trend in Fig 7 is that urbanization rate showed consistently positive associations across all four factors. Negative associations with GDP per capita and population density were concentrated mainly in strength and speed. Geographic and environmental covariates showed more domain-specific patterns.

**Strength.** The strength factor showed clear socioeconomic associations. Urbanization rate was positively associated with strength (β=0.684, 94% HDI [0.408,0.980]), indicating higher strength-related performance in more urbanized areas after adjustment for the other model components. In contrast, population density and GDP per capita were negatively associated with strength (population density: β=−0.383, 94% HDI [−0.632,−0.133]; GDP per capita: β=−0.285, 94% HDI [−0.531,−0.042]). Other geographic, climatic, and expenditure-related covariates showed weaker evidence, with 94% HDIs overlapping 0.

**Speed.** The speed factor exhibited a similar but more pronounced socioeconomic pattern. Urbanization rate was positively associated with speed (β=0.645, 94% HDI [0.404,0.904]). Population density and GDP per capita were negatively associated with speed (population density: β=−0.359, 94% HDI [−0.589,−0.123]; GDP per capita: β=−0.487, 94% HDI [−0.722,−0.260]). Annual mean relative humidity was also negatively associated with speed (β=−0.247, 94% HDI [−0.428,−0.088]). Annual precipitation, mean elevation, annual mean temperature, and culture/sports/media expenditure showed limited posterior support, with HDIs spanning 0.

**Endurance.** For endurance, urbanization rate was positively associated with the latent factor score (β=0.373, 94% HDI [0.031,0.712]). The remaining covariates had 94% HDIs overlapping 0, including population density, GDP per capita, precipitation, elevation, temperature, humidity, and culture/sports/media expenditure. This pattern suggests weaker or more uncertain covariate associations for endurance under the current data scale and model specification.

**Flexibility.** Flexibility showed a combined socioeconomic and topographic pattern. Urbanization rate was positively associated with flexibility (β=0.416, 94% HDI [0.155,0.662]). Mean elevation was also positively associated with flexibility (β=0.517, 94% HDI [0.035,0.988]). Other covariates, including population density, GDP per capita, precipitation, temperature, humidity, and culture/sports/media expenditure, showed weaker posterior support, with HDIs including 0.

Overall, Stage 2 revealed both common and domain-specific covariate patterns. Urbanization rate showed a consistent positive association with all four latent fitness factors. Negative associations with population density and GDP per capita were mainly observed for strength and speed. Environmental and geographic covariates showed more factor-specific patterns, including the negative association between relative humidity and speed and the positive association between elevation and flexibility. These estimates should be interpreted as conditional associations under the specified spatiotemporal model, rather than as causal effects.

### 3.3 Spatial effects and heterogeneity

After adjustment for covariates, educational-stage effects, and shared temporal trends, all four latent fitness factors still showed residual spatial structure. The spatial random effects $S_{i,k}$ were constrained to sum to zero ($\sum_{i}S_{i,k}=0$). Their posterior means can be interpreted as the adjusted deviation of city *i* from the provincial mean for factor *k*, conditional on the other model components.

**Fig 8:**
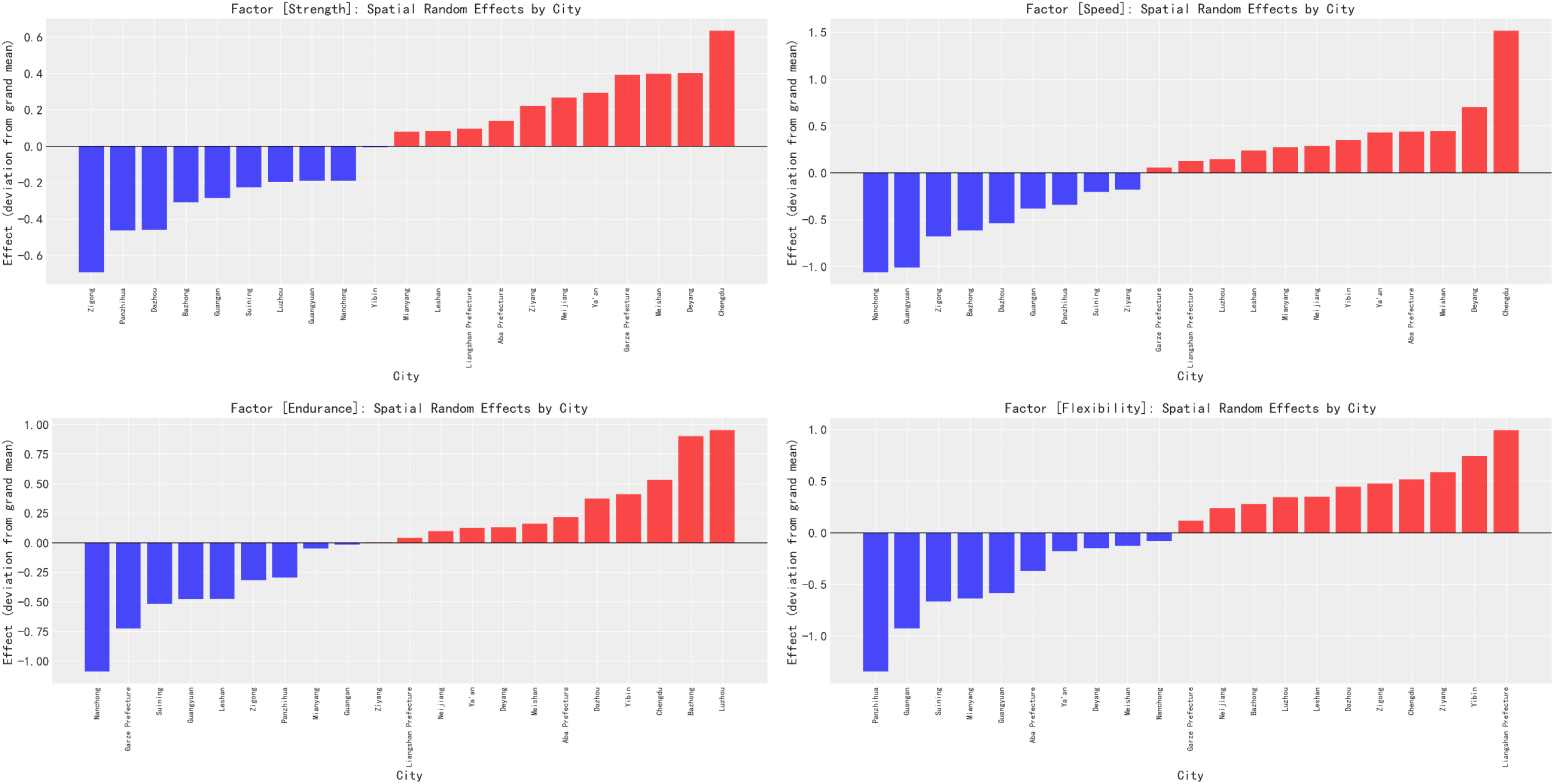
Posterior mean maps of spatial random effects.

**Fig 9:**
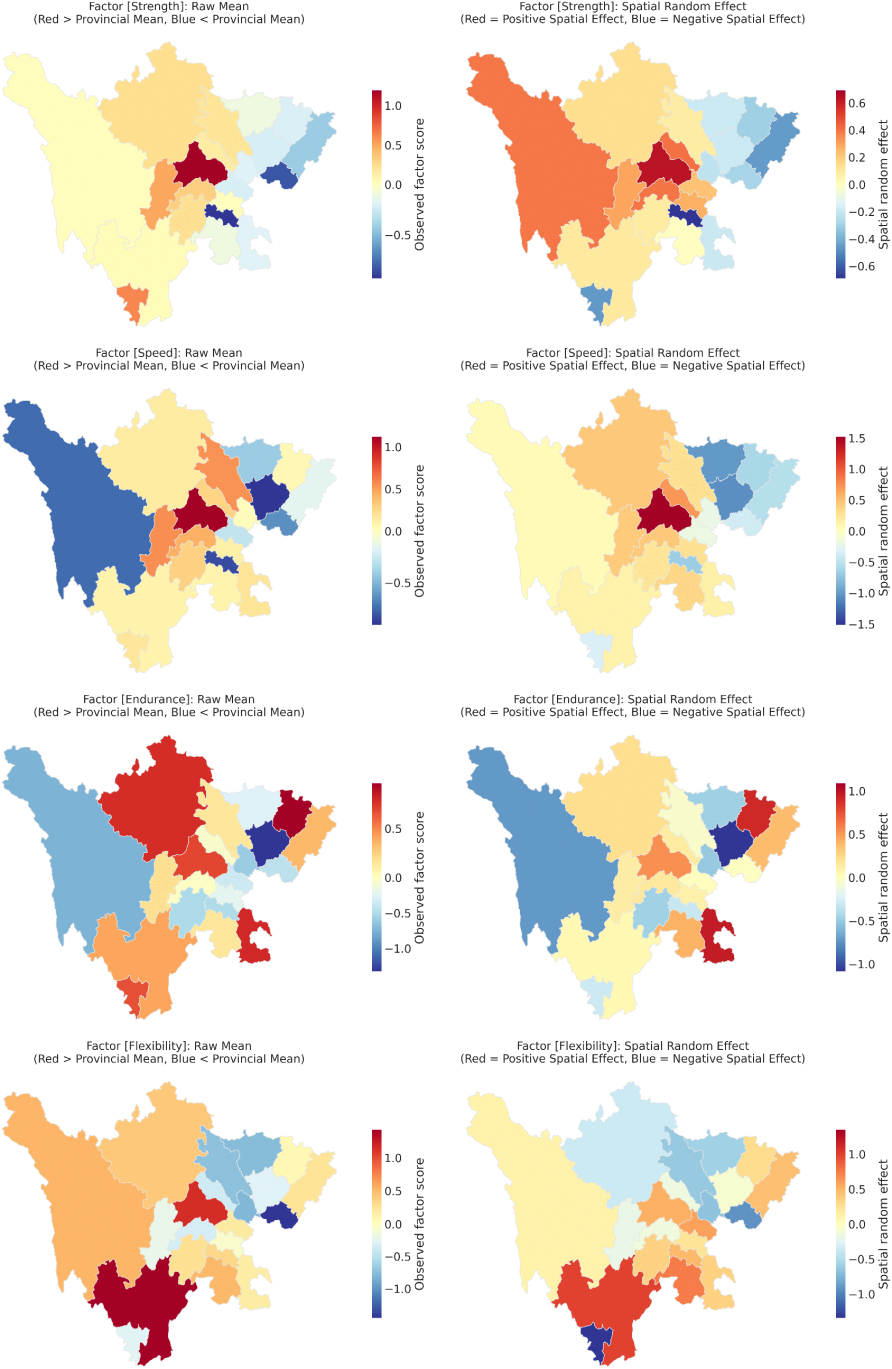
Comparison of raw mean factor levels and adjusted spatial random effects ($S_{i,k}$) for the four fitness factors. For each factor, the left panel shows the centered raw mean factor level (red indicates above the provincial mean and blue indicates below), and the right panel shows the posterior mean of the spatial random effect $S_{i,k}$ from the Stage 2 model (red indicates positive residual deviation and blue indicates negative residual deviation). Administrative boundary data were obtained from gbOpen under the CC BY 4.0 License. The data were subset to Sichuan Province and formatted by the authors for visualization.

Across cities, the posterior ranges of spatial effects were strength [−0.692, 0.635], speed [−1.061, 1.517], endurance [−1.087, 0.953], and flexibility [−1.344, 0.995]. As shown in Fig 8, residual spatial heterogeneity was present for all four factors, with stronger contrasts for speed and endurance. Fig 9 compares raw mean factor levels with adjusted spatial random effects. Raw means reflect the combined influence of observed covariates, temporal trends, and spatial structure, whereas $S_{i,k}$ captures residual spatial deviation after adjustment. The main trend is that raw regional averages and adjusted spatial effects do not always coincide. This comparison helps distinguish covariate-driven regional differences from persistent residual advantages or disadvantages, which may reflect unmeasured mechanisms such as resource allocation, training systems, accessibility, or testing organization.

**Strength.** The strength factor showed a coherent positive cluster around Chengdu. Chengdu had the largest positive deviation (*S*=0.635), and several adjacent cities also had positive deviations, including Deyang (0.402), Meishan (0.398), Ziyang (0.221), Neijiang (0.268), and Ya’an (0.295). In contrast, negative deviations were observed in Zigong (−0.692), Panzhihua (−0.463), Dazhou (−0.459), Bazhong (−0.307), and Guang’an (−0.283). These patterns indicate residual spatial clustering in strength after adjustment for observed covariates and temporal trends.

**Speed.** The spatial structure for speed showed the strongest contrast. Chengdu had the largest positive deviation (*S*=1.517), with nearby positive deviations in Deyang (0.700), Meishan (0.446), Ya’an (0.429), and Aba Prefecture (0.443). The strongest negative deviations occurred in Nanchong (−1.061) and Guangyuan (−1.013), with additional negative deviations in Bazhong (−0.615), Dazhou (−0.536), and Zigong (−0.676). This pattern suggests that speed-related performance retained strong residual spatial stratification.

**Endurance.** Endurance also showed substantial residual spatial heterogeneity, but its pattern differed from strength and speed. The largest positive deviations were observed in Luzhou (0.953) and Bazhong (0.902), followed by Chengdu (0.533), Yibin (0.412), and Dazhou (0.373). The largest negative deviation occurred in Nanchong (−1.087), with further negative deviations in Garze Prefecture (−0.725), Suining (−0.517), Guangyuan (−0.477), and Leshan (−0.475). The contrast between nearby areas, such as Bazhong and Nanchong, indicates factor-specific residual spatial variation in endurance.

**Flexibility.** Flexibility showed localized residual contrasts. Liangshan Prefecture had the largest positive deviation (0.995), followed by Yibin (0.744), Ziyang (0.587), Chengdu (0.515), Zigong (0.475), and Dazhou (0.444). Panzhihua had the largest negative deviation (−1.344), with additional negative deviations in Guang’an (−0.927), Suining (−0.668), Mianyang (−0.640), and Guangyuan (−0.586). These results suggest that flexibility had a more localized spatial pattern than strength and speed.

Overall, all four latent fitness factors exhibited residual spatial heterogeneity after adjustment for covariates, educational-stage effects, and temporal trends. Spatial clustering was particularly evident for strength and speed, while speed and endurance showed larger magnitudes of residual spatial variation. Comparing raw mean factor levels with adjusted spatial random effects helped distinguish covariate-driven regional differences from persistent residual deviations. This provides a practical basis for identifying regions that may require targeted follow-up.

### 3.4 Covariate collinearity and sensitivity analysis

**Fig 10:**
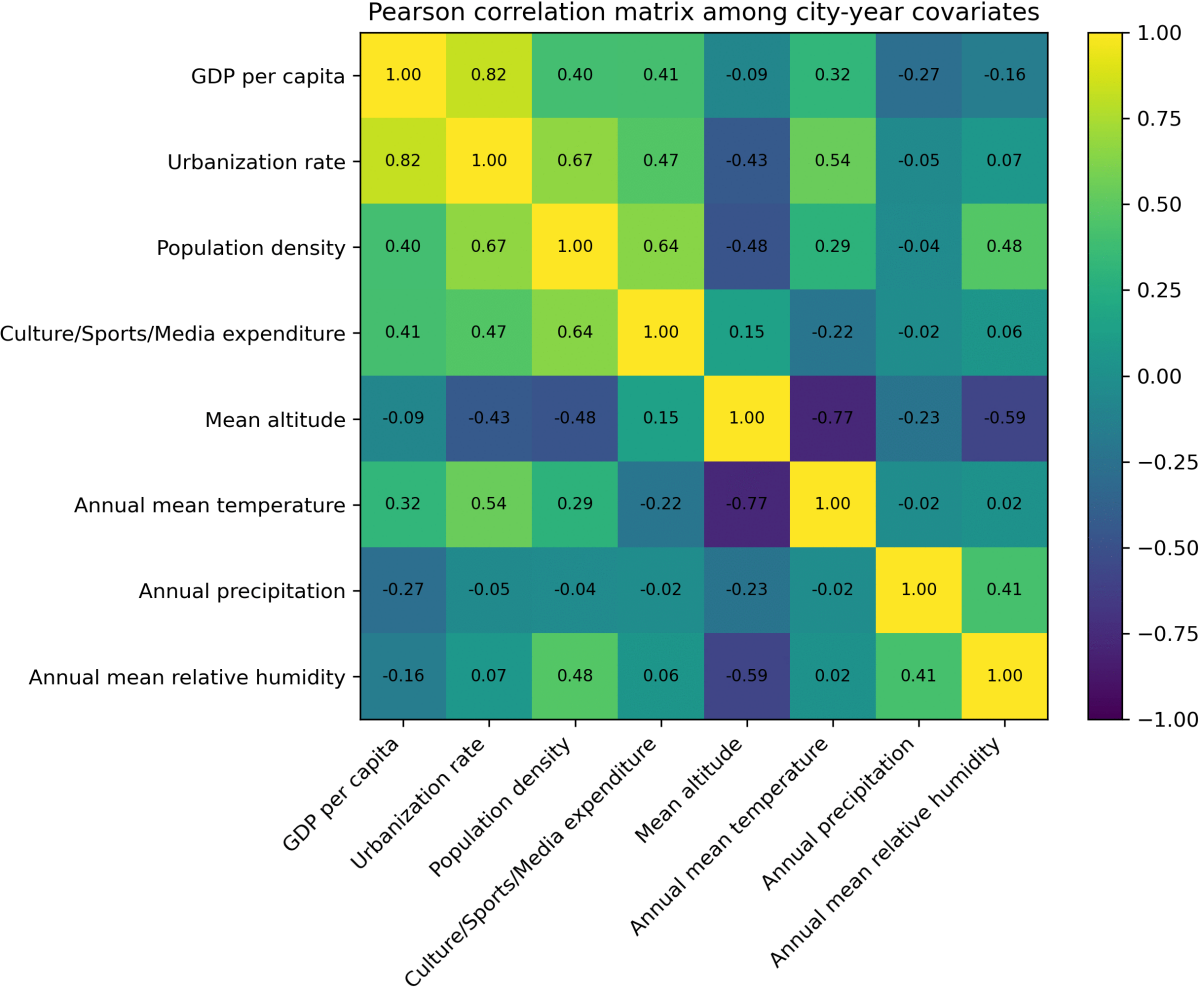
Pearson correlation matrix among city-year covariates.

To assess potential multicollinearity among the city-year covariates, we calculated the Pearson correlation matrix and variance inflation factors (VIFs). The correlation matrix is shown in Fig 10. The diagnostics indicated moderate to strong collinearity among several socioeconomic and geo-climatic variables. In particular, GDP per capita was strongly correlated with urbanization rate (*r* = 0.82), and urbanization rate was moderately correlated with population density (*r* = 0.67). Population density was also moderately correlated with culture/sports/media expenditure (*r* = 0.64). Among geo-climatic variables, mean altitude was strongly negatively correlated with annual mean temperature (r=−0.77).

We therefore conducted a covariate-inclusion sensitivity analysis for the main socioeconomic variables highlighted in the interpretation. Specifically, we refitted the Stage 2 models after excluding GDP per capita, population density, or urbanization rate one at a time. Table 5 summarizes the posterior means of the key socioeconomic coefficients under the full and reduced specifications. The positive association of urbanization rate was generally stable: after excluding GDP per capita or population density, the posterior mean of urbanization rate remained positive for all four latent factors. The negative associations of GDP per capita and population density with the speed factor were also stable across the reduced specifications. However, for strength and endurance, the GDP per capita coefficient changed direction and became close to zero after urbanization rate was removed, indicating sensitivity to correlated socioeconomic covariates.

**Table 5:** Covariate-inclusion sensitivity analysis for key socioeconomic coefficients.

Factor	Covariate	Full model	Drop GDP	Drop population density	Drop urbanization
Strength	Urbanization rate	0.684	0.485	0.560	–
	GDP per capita	−0.285	–	−0.276	0.078
	Population density	−0.383	−0.369	–	−0.195
Speed	Urbanization rate	0.645	0.445	0.541	–
	GDP per capita	−0.487	–	−0.503	−0.431
	Population density	−0.359	−0.369	–	−0.273
Endurance	Urbanization rate	0.373	0.292	0.314	–
	GDP per capita	−0.144	–	−0.140	0.014
	Population density	−0.176	−0.175	–	−0.079
Flexibility	Urbanization rate	0.416	0.364	0.363	–
	GDP per capita	−0.160	–	−0.174	−0.030
	Population density	−0.170	−0.188	–	−0.064

Table notes: Values are posterior means of standardized covariate coefficients from the Stage 2 models. “Drop GDP”, “Drop population density”, and “Drop urbanization” denote reduced specifications in which GDP per capita, population density, or urbanization rate was excluded, respectively.

Table 6 summarizes the stability of posterior spatial effects under the reduced specifications. The correlations between the full-model and reduced-model spatial effects ranged from 0.759 to 0.990 across factors and sensitivity models. Taken together, the sensitivity analyses suggest that the spatial heterogeneity patterns are relatively robust to alternative socioeconomic covariate specifications. However, some individual socioeconomic coefficients, especially GDP per capita for strength and endurance, are sensitive to the inclusion of correlated covariates. Therefore, the covariate effects should be interpreted as conditional ecological associations within the specified multivariable model rather than as independent causal effects.

**Table 6:** Correlations of posterior spatial effects between full and reduced models.

Factor	Drop GDP	Drop population density	Drop urbanization
Strength	0.933	0.888	0.759
Speed	0.878	0.950	0.872
Endurance	0.990	0.985	0.949
Flexibility	0.989	0.987	0.930

Table notes: Values are Pearson correlations between posterior mean spatial random effects from the full model and those from the reduced models. Higher values indicate more stable spatial heterogeneity patterns under alternative socioeconomic covariate specifications.

### 3.5 PSIS-LOO comparison

To assess the empirical contribution of spatial structure, we compared the full spatiotemporal model with a no-spatial model for each latent fitness factor. The full model included both spatial and temporal components, whereas the no-spatial model removed the spatial random effect $S_{i,k}$. Model comparison was based on the expected log pointwise predictive density estimated by Pareto-smoothed importance sampling leave-one-out cross-validation (PSIS-LOO), denoted *elpd*_loo_. Larger values indicate better leave-one-out predictive performance. We report $\Delta elpd_{\text{loo}}$, its standard error, PSIS-LOO stacking weights, and Pareto-*k* diagnostics in Table 7.

**Table 7:** PSIS-LOO comparison between the full model and the no-spatial model.

Factor	Model	$elpd_{loo}$	se(elpd)	$p_{loo}$	$\Delta elpd_{loo}$	Weight	$k_{\max}$
Strength	Full model	−578.31	30.25	35.05	0.00	0.9906	0.5567
	No-spatial model	−631.15	25.82	22.27	52.84	0.0094	0.4397
Speed	Full model	−375.94	21.77	35.29	0.00	0.9773	0.4248
	No-spatial model	−550.93	18.49	18.20	174.99	0.0227	0.2734
Endurance	Full model	−606.96	24.73	35.27	0.00	0.9870	0.3534
	No-spatial model	−745.34	22.80	18.94	138.38	0.0130	0.3436
Flexibility	Full model	−363.51	19.70	34.81	0.00	1.0000	0.4002
	No-spatial model	−573.82	18.95	17.07	210.32	0.0000	0.2873

Table notes: *elpd*_loo_ denotes the expected log pointwise predictive density estimated by PSIS-LOO. *p*_loo_ is the effective number of parameters. $\Delta elpd_{\text{loo}}$ is computed relative to the best-performing model within each fitness factor. “Weight” refers to the PSIS-LOO stacking weight. $k_{\max}$ is the maximum Pareto-*k* diagnostic value.

Across all four factors, the full model had higher PSIS-LOO predictive performance than the no-spatial model. The improvements in *elpd*_loo_ were 52.84 for strength, 174.99 for speed, 138.38 for endurance, and 210.32 for flexibility. The stacking weights were close to 1 for the full models, indicating that the spatial models were consistently favored under this comparison criterion. As expected, the effective number of parameters *p*_loo_ was larger for the full model than for the no-spatial model, reflecting the additional spatial random effects.

The Pareto-*k* diagnostics did not indicate highly influential observations under the fitted models. This suggests that the PSIS approximation was stable for the present model comparisons. Together with the spatial-effect maps, these results indicate that residual spatial structure contributed meaningful predictive information for all four latent fitness factors.

Overall, the full spatiotemporal model consistently outperformed the no-spatial model across all four latent fitness factors. The largest improvements were observed for flexibility and speed, followed by endurance and strength. These results support the inclusion of spatial random effects in the factor-level surveillance models, while the Pareto-*k* diagnostics supported the stability of the PSIS-LOO comparisons. Given the spatial and temporal dependence in the data, however, standard pointwise PSIS-LOO should be interpreted as evidence of improved conditional pointwise predictive fit rather than fully independent spatial or temporal generalization.

## 4 Discussion

This study proposes a two-stage Bayesian analytical framework for provincial adolescent physical fitness surveillance. The framework combines a latent measurement model with factor-level spatiotemporal modeling. It is designed to characterize multidimensional fitness structure, conditional covariate associations, and residual spatial heterogeneity.

The results support four main findings. First, the Bayesian CFA in Stage 1 provided an interpretable mapping from multiple test items to four latent fitness factors: strength, speed, endurance, and flexibility. Second, the Bayesian spatiotemporal model in Stage 2 identified residual spatial structure after adjustment for observed covariates, educational-stage effects, and temporal trends. Third, PSIS-LOO model comparison favored models with spatial random effects over corresponding no-spatial models across all four factors in terms of conditional pointwise predictive fit. Fourth, comparing raw mean factor levels with adjusted spatial random effects helped distinguish observed regional differences from persistent residual deviations under the fitted model. Together, these results show that separating latent measurement from regional spatiotemporal modeling can improve the interpretability of provincial fitness surveillance.

### 4.1 Key findings

First, the four-factor structure showed clear interpretability across educational stages. Compared with approaches based on single indicators or simple score aggregation, latent factor modeling accounts for inter-item correlations, measurement error, and domain-specific fitness information. This provides a more stable basis for regional comparison at the factor level.

Second, the covariate results showed both common and domain-specific patterns. Urbanization rate showed a positive conditional association with all four latent fitness factors under the specified multivariable model. In contrast, negative conditional associations with population density and GDP per capita were mainly observed for strength and speed. Environmental and geographic covariates showed more factor-specific associations. For example, annual mean relative humidity was negatively associated with speed, whereas mean elevation was positively associated with flexibility. These results should be interpreted as conditional ecological associations rather than causal effects, because they are based on aggregated regional data and may be affected by residual confounding and multicollinearity among city-level covariates.

Third, the spatial random effects indicated residual regional heterogeneity. After adjustment for covariates, educational-stage effects, and temporal trends, all four fitness factors still exhibited spatial deviations. Spatial clustering was particularly evident for strength and speed, while speed and endurance showed larger magnitudes of residual spatial variation. These patterns suggest that neighboring cities may share unmeasured regional characteristics, such as resource allocation, school physical education organization, accessibility, or testing implementation. These interpretations are hypothesis-generating and should not be read as causal explanations of the observed spatial patterns.

Fourth, PSIS-LOO model comparison supported the inclusion of spatial random effects. Across all four factors, the full spatiotemporal model outperformed the corresponding no-spatial model under the pointwise PSIS-LOO criterion. Pareto-*k* diagnostics did not indicate unstable importance sampling. These findings suggest that residual spatial structure contains useful information for conditional pointwise prediction within the observed spatiotemporal panel. However, because observations are spatially and temporally correlated, standard pointwise PSIS-LOO should not be interpreted as fully independent validation for new cities, years, or regions.

### 4.2 Interpretation and potential mechanisms

The observed patterns may be interpreted through three broad perspectives: resource provision, behavioral opportunity, and environmental constraint. These interpretations are exploratory and should not be viewed as causal conclusions.

Urbanization may be associated with better access to school sports facilities, qualified physical education teachers, extracurricular training, public services, transportation infrastructure, and organized sports activities. These factors may improve both the frequency and quality of physical training. This may help explain the positive associations between urbanization rate and the four latent fitness factors. However, urbanization may also be related to sedentary behavior, screen exposure, dietary changes, and academic pressure. Therefore, the observed positive associations should be interpreted as net conditional associations under the current covariate set, rather than as evidence that urbanization itself improves fitness.

The negative associations of population density and GDP per capita with strength and speed may have several explanations. High-density areas may have limited outdoor space and more constrained daily schedules. Economic development may not necessarily translate into higher youth sports participation, especially when academic pressure, extracurricular tutoring, or sedentary lifestyles are also present. Differences in testing organization and participation patterns may also affect regional score distributions. These explanations remain hypotheses and require more detailed school- or individual-level data for evaluation.

The negative association between annual mean relative humidity and speed may be related to exercise comfort and outdoor activity opportunities. Hot and humid conditions may reduce the frequency or sustainability of high-intensity outdoor activity. The positive association between elevation and flexibility may reflect local lifestyle factors, ethnic or cultural sports traditions, training content, or test familiarity. Because environmental covariates may be correlated with socioeconomic and regional characteristics, these findings should be viewed as domain-specific signals rather than definitive mechanisms.

### 4.3 Comparison with existing literature

Previous studies on adolescent physical fitness surveillance often rely on single indicators, such as endurance runs or BMI, or on composite scores for regional comparison. These approaches may be affected by measurement error, weighting choices, and dimensional mixing. Latent variable methods, including CFA and SEM, provide a structured alternative by attributing shared variation across test items to latent fitness dimensions while separating measurement error from structural relationships [23,24]. The present study extends this approach by using Bayesian CFA as the measurement stage and then modeling the resulting factor-level outcomes in a spatiotemporal framework [9].

In public health and spatial epidemiology, CAR and BYM-type spatial random effects are widely used to capture unobserved spatially correlated risk [11,25]. The spatial effect maps in this study show that adolescent fitness factors also contain residual spatial structure after adjustment for observed covariates and temporal trends. This suggests that tools from disease mapping, such as exceedance probabilities, regional prioritization, and uncertainty visualization, may be useful for physical fitness surveillance.

PSIS-LOO was used to compare models with and without spatial random effects. Pareto-*k* diagnostics did not indicate unstable importance sampling under the fitted models. This use of PSIS-LOO follows contemporary recommendations for Bayesian model evaluation, where predictive criteria are combined with diagnostic checks [22,26,27]. At the same time, because the observations are spatially and temporally correlated, the PSIS-LOO results should be interpreted as comparative evidence within the fitted modeling framework rather than as fully independent validation.

### 4.4 Practical implications

The proposed framework provides a statistical basis for provincial physical fitness surveillance. It can help summarize multidimensional test results, compare regions at the factor level, and identify areas with persistent residual deviations after adjustment for observed covariates and temporal trends.

By characterizing strength, speed, endurance, and flexibility separately, the framework allows policy discussion to move beyond overall score comparisons. Regions can be examined according to specific fitness domains, while also considering covariate patterns and posterior uncertainty.

The posterior mean of the spatial random effect $S_{i,k}$ represents the adjusted residual deviation of city *i* for factor *k*. Regions with high raw means but weak or negative $S_{i,k}$ values may have favorable observed conditions but limited residual advantage after adjustment. Conversely, regions with low raw means but positive $S_{i,k}$ values may have better-than-expected adjusted performance. These contrasts can guide follow-up investigation into local practices, resource allocation, or testing implementation.

A practical surveillance strategy may combine raw mean levels, adjusted spatial effects, and posterior uncertainty, such as HDIs or exceedance probabilities. This can support transparent regional ranking and benchmarking, consistent with spatial public health risk allocation frameworks [25]. Such information may help identify regions for further monitoring or targeted support, while avoiding decisions based only on raw averages.

For highly urbanized regions, policy discussion may focus on whether favorable resources are balanced against sedentary behavior, academic pressure, and limited activity time. Interventions may emphasize daily activity opportunities, safe active transport, access to open spaces, and sedentary behavior reduction. These directions are consistent with evidence on the built environment, activity opportunities, and physical activity [28,29].

For resource-limited regions, the results may support attention to basic capacity building. Relevant areas include physical education time, equipment and facility provision, teacher training, after-school sports services, and integration with nutrition and health education. These priorities are consistent with the principles of Quality Physical Education [30].

For regions with topographic or climatic constraints, surveillance results may help inform context-sensitive strategies. These may include indoor activity options, season-specific training plans, load management, and monitoring of endurance-related dimensions. Such strategies align with global frameworks that emphasize expanding activity opportunities and reducing environmental barriers [29].

For regions with high uncertainty or abrupt spatial contrasts, cautious interpretation is needed. Pilot interventions, intensified monitoring, and rolling model updates can help stabilize evidence before large-scale implementation.

Overall, the framework integrates factor-level diagnosis, residual spatial effect estimation, uncertainty quantification, and model comparison. It provides a practical basis for differentiated monitoring and resource allocation at the provincial scale. It can also be extended in future work to spatially varying coefficient models or multiscale mechanism analysis.

## 5 Conclusions

This study presents a two-stage Bayesian framework for provincial adolescent physical fitness surveillance. In Stage 1, Bayesian CFA maps multiple test items across educational stages onto four interpretable latent fitness factors. In Stage 2, Bayesian spatiotemporal hierarchical regression models factor-level outcomes to estimate conditional covariate associations, temporal trends, and residual spatial heterogeneity. By separating latent measurement from regional spatiotemporal modeling, the framework provides a more interpretable basis for regional comparison, covariate assessment, and spatial diagnosis.

Three main conclusions emerge. First, the four latent fitness factors showed clear and interpretable residual spatial structure at the provincial scale. The spatial random effects $S_{i,k}$ identified cities that remained above or below the provincial mean after adjustment for observed covariates, educational-stage effects, and shared temporal trends. These adjusted deviations provide useful evidence for regional comparison and follow-up investigation, rather than direct evidence of specific causal mechanisms.

Second, model comparison supported the inclusion of spatial random effects. Across all four factors, the full spatiotemporal model outperformed the corresponding no-spatial model under the pointwise PSIS-LOO criterion, with stable Pareto-*k* diagnostics. This suggests that residual spatial structure contains useful information for conditional pointwise prediction within the observed spatiotemporal panel. Because the analytical units are spatially and temporally correlated, these results should not be interpreted as fully independent validation for new cities, years, or regions.

Third, comparing raw mean factor levels with adjusted spatial random effects helped distinguish observed regional differences from residual deviations under the fitted model. This comparison can reduce the risk of relying only on raw averages and can help identify regions that warrant further monitoring or local investigation. The resulting regional evidence should be interpreted as surveillance-oriented and hypothesis-generating, not as causal attribution of regional fitness differences.

**Limitations and future directions.** The results should be interpreted in light of several limitations. First, the analysis was conducted at the city/prefecture level using aggregated data. Therefore, the findings describe regional patterns and cannot be directly extrapolated to individual-level mechanisms. Future work could use multilevel models to distinguish regional, school-level, and individual effects and to assess whether the findings are robust at finer spatial scales.

Second, the present study used a two-stage modeling strategy. Although uncertainty from Stage 1 was propagated to Stage 2 through cell-level posterior uncertainty measures, a fully Bayesian joint model could estimate the measurement and spatiotemporal components simultaneously. Such an extension may provide more coherent uncertainty propagation, although it would also increase model complexity.

Third, the current covariate set mainly includes socioeconomic, geographic, and climatic characteristics. It does not include more proximal mechanisms, such as school sports resources, physical education curriculum time, extracurricular participation, academic pressure, policy implementation, or testing organization. Including these variables in future studies would improve the interpretation of regional differences.

Fourth, standard pointwise PSIS-LOO has limitations under spatial and temporal dependence. Because nearby cities and repeated yearly observations may not be fully independent, PSIS-LOO may overstate predictive generalization to entirely new cities, years, or regions. Therefore, the LOO results in this study should be interpreted as evidence of improved conditional pointwise predictive fit within the observed spatiotemporal panel. Future work could use grouped or blocked validation, such as leave-one-city-out, leave-one-year-out, or leave-one-region-out cross-validation.

Future methodological extensions may incorporate richer nonlinear, interaction, and accessibility-oriented structures. Examples include spline- or Gaussian process-based temporal trends, threshold effects of urbanization or environmental variables, and interactions between resource provision and educational stage [31,32]. Future work could also include indicators of access to school sports facilities, public sports venues, green spaces, health services, and transportation convenience to better explain residual regional differences in adolescent fitness factors. Such extensions would connect adolescent fitness surveillance with broader accessibility and equity perspectives used in regional public service evaluation [33]. In addition, multivariate spatial models could be developed to capture shared and factor-specific spatial mechanisms across the four fitness dimensions [34]. Together, these extensions would further improve the analysis of multidimensional adolescent physical fitness surveillance.

## References

[1] Ortega FB, Ruiz JR, Castillo MJ, Sjöström M. Physical fitness in childhood and adolescence: a powerful marker of health. Int J Obes (Lond). 2008;32(1):1–11. doi: 10.1038/sj.ijo.0803774 18043605

[2] Ruiz JR, Sui X, Lobelo F, Morrow JR Jr, Jackson AW, Sjöström M, et al. Association between muscular strength and mortality in men: prospective cohort study. BMJ. 2008;337(7661):a439. doi: 10.1136/bmj.a439 18595904 PMC2453303

[3] Guthold R, Stevens GA, Riley LM, Bull FC. Global trends in insufficient physical activity among adolescents: a pooled analysis of 298 population-based surveys with 1·6 million participants. Lancet Child Adolesc Health. 2020;4(1):23–35. doi: 10.1016/S2352-4642(19)30323-2 31761562 PMC6919336

[4] Tomkinson GR, Lang JJ, Tremblay MS. Temporal trends in the cardiorespiratory fitness of children and adolescents representing 19 high-income and upper middle-income countries between 1981 and 2014. Br J Sports Med. 2019;53(8):478–86. doi: 10.1136/bjsports-2017-097982 29084727

[5] Chaput J-P, Willumsen J, Bull F, Chou R, Ekelund U, Firth J, et al. 2020 WHO guidelines on physical activity and sedentary behaviour for children and adolescents aged 5-17 years: summary of the evidence. Int J Behav Nutr Phys Act. 2020;17(1):141. doi: 10.1186/s12966-020-01037-z 33239009 PMC7691077

[6] Gao E. Spatial–temporal trends in global childhood overweight and obesity from 1975 to 2030: a weight mean center and projection analysis of 191 countries. Global Health. 2023. doi: 10.1186/s12992-023-00954-5 PMC10403851 37542334

[7] Tong Y, Zhang X, Yu H, Jia P, Hou L, Kong Y. Spatiotemporal evolution of overweight and obesity among Chinese adolescents from 2016 to 2020. iScience. 2023;26(11):108742. doi: 10.1016/j.isci.2023.108742 PMC10790006 38230263

[8] Blangiardo M, Cameletti M. Spatial and spatio-temporal Bayesian models with R-INLA. John Wiley & Sons; 2015.

[9] Lee SY. Structural equation modeling: A Bayesian approach. John Wiley & Sons; 2007.

[10] Muthén B, Asparouhov T. Bayesian structural equation modeling: a more flexible representation of substantive theory. Psychol Methods. 2012;17(3):313–35. doi: 10.1037/a0026802 22962886

[11] Besag J, York J, Mollie A. Bayesian image restoration, with two applications in spatial statistics. Ann Inst Stat Math. 1991;43(1):1–20. doi: 10.1007/bf00116466

[12] Riebler A, Sørbye SH, Simpson D, Rue H. An intuitive Bayesian spatial model for disease mapping that accounts for scaling. Stat Methods Med Res. 2016;25(4):1145–65. doi: 10.1177/0962280216660421 27566770

[13] Rue H, Martino S, Chopin N. Approximate Bayesian Inference for Latent Gaussian models by using Integrated Nested Laplace Approximations. J R Stat Soc Ser B Stat Methodol. 2009;71(2):319–92. doi: 10.1111/j.1467-9868.2008.00700.x

[14] Gelfand AE, Kim H-J, Sirmans CF, Banerjee S. Spatial Modeling With Spatially Varying Coefficient Processes. J Am Stat Assoc. 2003;98(462):387–96. doi: 10.1198/016214503000170 39421645 PMC11484471

[15] Finley AO. Comparing spatially‐varying coefficients models for analysis of ecological data with non‐stationary and anisotropic residual dependence. Methods Ecol Evol. 2010;2(2):143–54. doi: 10.1111/j.2041-210x.2010.00060.x

[16] Song C, Shi X, Wang J. Spatiotemporally Varying Coefficients (STVC) model: a Bayesian local regression to detect spatial and temporal nonstationarity in variables relationships. Ann GIS. 2020;26(3):277–91. doi: 10.1080/19475683.2020.1782469

[17] Simpson D, Rue H, Riebler A, Martins TG, Sørbye SH. Penalising Model Component Complexity: A Principled, Practical Approach to Constructing Priors. Statist Sci. 2017;32(1). doi: 10.1214/16-sts576

[18] Gómez MJ, Barboza LA, Vásquez P, Moraga P. Bayesian spatial modeling of childhood overweight and obesity prevalence in Costa Rica. BMC Public Health. 2023;23(1):651. doi: 10.1186/s12889-023-15486-1 37016373 PMC10074779

[19] Brunsdon C, Fotheringham AS, Charlton ME. Geographically Weighted Regression: A Method for Exploring Spatial Nonstationarity. Geogr Analys. 1996;28(4):281–98. doi: 10.1111/j.1538-4632.1996.tb00936.x

[20] Fotheringham AS, Brunsdon C, Charlton M. Geographically Weighted Regression: The Analysis of Spatially Varying Relationships. Chichester: Wiley; 2002.

[21] Fotheringham AS, Yang W, Kang W. Multiscale Geographically Weighted Regression (MGWR). Ann Am Assoc Geogr. 2017;107(6):1247–65. doi: 10.1080/24694452.2017.1352480

[22] Vehtari A, Gelman A, Gabry J. Practical Bayesian model evaluation using leave-one-out cross-validation and WAIC. Stat Comput. 2017;27(5):1413–32. doi: 10.1007/s11222-016-9696-4

[23] Bollen KA. Structural equations with latent variables. John Wiley & Sons; 1989.

[24] Brown TA. Confirmatory factor analysis for applied research. Guilford Publications; 2015.

[25] Lawson AB. Bayesian disease mapping: hierarchical modeling in spatial epidemiology. 3rd ed. Boca Raton: CRC Press; 2018.

[26] Vehtari A, Simpson D, Gelman A, Yao Y, Gabry J. Pareto smoothed importance sampling. J Mach Learn Res. 2024;25:1–58.41334350

[27] Watanabe S. Asymptotic equivalence of Bayes cross validation and widely applicable information criterion in singular learning theory. J Mach Learn Res. 2010;11:3571–94.

[28] Sallis JF, Cerin E, Conway TL, Adams MA, Frank LD, Pratt M, et al. Physical activity in relation to urban environments in 14 cities worldwide: a cross-sectional study. Lancet. 2016;387(10034):2207–17. doi: 10.1016/S0140-6736(15)01284-2 27045735 PMC10833440

[29] World Health Organization. Global Action Plan on Physical Activity 2018–2030: More active people for a healthier world. Geneva: WHO. World Health Organization; 2018.

[30] UNESCO. Quality physical education (QPE): Guidelines for policy-makers. Paris: UNESCO. United Nations Educational, Scientific and Cultural Organization; 2015.

[31] Cressie N, Wikle CK. Statistics for spatio-temporal data. John Wiley & Sons; 2011.

[32] Banerjee S, Carlin BP, Gelfand AE. Hierarchical Modeling and Analysis for Spatial Data. 2nd ed. Boca Raton: CRC Press; 2014.

[33] Yang Z, Guo Y, Feng X, Zhou Y, Zhou P, Li X, et al. Evaluating temporal variations in access to multi-tier hospitals using personal vehicles and public transit: Implications for healthcare equity. Sustain Cities Soc. 2024;113:105687. doi: 10.1016/j.scs.2024.105687

[34] Gelfand AE, Banerjee S. Multivariate Spatial Process Models. In: Gelfand AE, Diggle P, Fuentes M, Guttorp P, editors. Handbook of Spatial Statistics. Boca Raton: CRC Press; 2010. p. 495–515.

